# Synthesis and Rational design of Europium and Lithium Doped Sodium Zinc Molybdate with Red Emission for Optical Imaging

**DOI:** 10.1038/s41598-019-38787-1

**Published:** 2019-02-21

**Authors:** Neha Jain, Ruchi Paroha, Rajan K. Singh, Siddhartha K. Mishra, Shivendra K. Chaurasiya, R. A. Singh, Jai Singh

**Affiliations:** 1 Department of Physics, Dr. Harisingh Gour Central University, Sagar, 470003 India; 2 Host-Pathogen Interaction and Signal Transduction Laboratory, Department of Microbiology, Dr. Harisingh Gour Central University, Sagar, 470003 India; 3 Department of Zoology, Dr. Harisingh Gour Central University, Sagar, 470003 India; 4 Department of Biological Science & Engineering, Maulana Azad National Institute of Technology, Bhopal, MP 462003 India

## Abstract

Highly efficient fluorescent and biocompatible europium doped sodium zinc molybdate (NZMOE) nanoprobes were successfully synthesized via Polyol method. Non-radiative defect centres get reduced with Li^+^ co-doping in NZMOE nanoprobes. XRD spectra and Rietveld refinement confirmed successful incorporation of lithium ion and crystallinity was also improved with Li^+^ co-doping. The shape of phosphor is rod shaped, as determined by TEM. Significant enhancement in photoluminescence intensity was observed with 266, 395 and 465 nm excitations. Profound red emission was recorded for 5 at% Li^+^ co-doped NZMOE nanoprobes with 266 nm excitation. It shows high asymmetry ratio (~15), color purity (94.90%) and good quantum efficiency (~70%). Judd Ofelt parameters have been calculated to measure intensity parameters and radiative transition rates. In order to measure biocompatibility of the nanoprobes, cytotoxicity assays were performed with HePG2 cells. The fluorescence emitted from phosphor material treated HePG2 cells was also measured by Laser Scanning Confocal Microscopy. The bright red fluorescence in HePG2 cells treated with very low concentration (20 μg/ml) of phosphor material indicates that it could be a promising phosphor for biological detection or bio-imaging.

## Introduction

Lanthanide activated fluorescent nano-phosphors have attracted enormous attention in optical storage, field emission display, solar cells, light emitting diodes, display technology, for night vision applications etc.^1–4^. It has broad range of photoluminescence (PL) emission depending upon type of transition *viz* 4f-4f and 4f-5d transition. Meanwhile, on the other hand lanthanides (Ln^3+^) have interesting properties such as good biocompatibility, low photo-bleaching and better photo-stability which makes it a promising material for drug delivery, cancer cells detection, multimode imaging etc.^5–7^. Nonetheless, Ln^3+^ ion needs a host which could provide energy to Ln^3+^ ion so that its parity forbidden 4f-4f transition has been allowed by host crystal field strength^8,9^. Continuing it, several hosts like NaYF_4_, NaGdF_4_, KGdF_4_, NaScF_4_, NaYbF_4_, NaLuF_4_, LaF_3_ etc. have been developed for bio-imaging purpose^10–15^. In addition, these hosts are bounded by stability at room temperature in ambient condition. However, oxides are not affected by aforementioned limitations due to their high chemical and thermal stability as well as their environment friendly nature^16^. There are several categories of oxide hosts such as molybdate, tungstate, phosphate, gallate and aluminate. In contemporary, oxide host has drawn much attention of the researchers especially for biomedical diagnosis, bio-imaging and drug-delivery etc. due to their bio-compatible properties^17–20^. In addition, for bio-imaging purpose the red fluorescence is suitable because of low scattering and good penetration depth to tissue etc^5,12^. Eu^3+^ is a well known lanthanide which provides strong red emission by electric dipole transition^16,17^.

Among various oxides, ZnMoO_4_ has aroused great interest due to its excellent PL emission properties, photo-catalytic activity, improved photo-stability and electronic conductivity^20–22^. ZnMoO_4_ has two phases, monoclinic and triclinic, depending upon the synthesis and annealing temperatures^23–25^. In the literature, PL properties of Eu^3+^ doped triclinic ZnMoO_4_ are reported for its various concentrations^26–28^. Nevertheless, to the best of our knowledge and available literature, PL properties of lanthanide activated sodium zinc molybdate have not been reported earlier. It belongs to the family of double molybdates and it has a large absorption cross section in the ultraviolet region. Therefore, it efficiently absorbs and transfer energy to doped Ln^3+^ ion.

As Eu^3+^ possessing main characteristic emission at 590 and 613 nm are caused due to electric and magnetic dipole transitions, both the transitions depend on crystal site symmetry and local environment around Eu^3+^ ion. The site symmetry of Eu^3+^into crystal can be designed by using Judd-Ofelt parameter Ω_j_ (j = 2, 4, 6)^29^. Higher Ω_2_ indicate, deep red emission which increases Li^+^ co-doping^30,31^. As, in bio-imaging, mostly red phosphor is used because scattering of red colour is minimum. Also biological performance of Eu^3+^ activated double molybdates rarely investigated. So, if Eu^3+^ activated sodium zinc molybdate is biocompatible then it can be a promising phosphor for bio-imaging purpose. Luitel *et al*. have studied up-conversion bio- imaging by Tm^3+^ and Yb^3+^ co-doped ZnMoO_4_ ^20^. However, cytotoxicity of monoclinic sodium zinc molybdate is not reported till date.

Since Eu^3+^ replaces Zn^2+^ ion in the crystal because of their similar atomic radii and causes charge imbalance problem, consequently, the defect centres are created. PL emission intensity reduces and non-radiative relaxation increases because of such types of quenching or defect centres. Although, such problems can be resolved by co-doping with alkali metals (Li^+^, Na^+^ and K^+^) which act as charge compensator. Xie *et al*. reported improved PL emission by Eu^3+^activated ZnMoO_4_ with charge compensation by alkali metals and found prominent emission for Li^+^ ion co-doped phosphor^28^. The reason behind it is that Li^+^ ion has smaller atomic radius, it easily enters into the crystal and affects crystal environment as well as the site symmetry of Eu^3+^ ion. However, Li^+^ concentration on ZnMoO_4_:Eu^3+^ was not optimized by them.

In this work, 2 at.% Eu^3+^ doped sodium zinc molybdate (NZMOE) have been prepared by Polyol route and co-doped with *x* at.% Li^+^ (*x*- 0, 2, 5, 7, 10)^32^. The phase of as prepared sample has been analysed by XRD which is monoclinic. Various vibrational modes of Mo-O bonds have been analysed by Raman spectra. The morphology and compositional analysis have been done by SEM (scanning electron microscopy), TEM (transmission electron microscopy) and EDX (energy dispersive X-ray analysis) analysis. PL emission and excitation spectra show variations in emission with Li^+^ co-doping. The CIE chromaticity co-ordinate shows color tuning (red-pink-yellow) at various excitation and Li^+^ incorporation. The colour purity and CCT values also calculated and it is high enough for as prepared Li^+^ co-doped sample. Cytotoxicity has been performed on HePG2 cells by WST assay.

## Experimental Section

### Synthesis of sodium zinc molybdate

Starting materials used in synthesis were Zn(NO_3_)_2_.6H_2_O (Merck 99.8%), Na_2_MoO_4_.2H_2_O (Merck 99.8%), Eu_2_O_3_ (Merck 99.9%), LiOH (Merck 99.5%), ethylene glycol (Merck) and urea (Himedia). At first, a solution of Europium nitrate was prepared by dissolving Eu_2_O_3_ in de-ionised water with adding of nitric acid, then add Zn(NO_3_)_2_.6H_2_O and LiOH. Access of nitric acid was removed by heating the solution at 80 °C with the addition of de-ionised water. In the above solution, ethylene glycol was added and the pH of solution was adjusted to 9 using urea, and then stirred for about one hour at 80 °C. In next step, 10 mL solution of Na_2_MoO_4_.2H_2_O was added and the white precipitate appeared. This solution was transferred in a round bottom flask and heated at 120 °C with continuous stirring for completion of reaction. The precipitate solution was kept undisturbed until the precipitate is settled down and the residual solution was removed. The precipitate washed out by de-ionised water, methanol and acetone to remove the remaining impurities. This precipitate is dried at 60 °C in an oven for 2 hrs to obtain final powder. This procedure is repeated for synthesis of Li^+^ co-doped (0, 2, 5, 7 and 10 at.%) and 2 at% Eu^3+^ doped sample. Here 2 at% Eu^3+^ doped sodium zinc molybdate (Na(OH)Zn_2_(MoO_4_)_2_.2.5H_2_O (NZMO)) is abbreviated as NZMOE.

### Maintenance of culture

HepG2 cell line (originally acquired from the American Type Culture Collection, USA) were cultured in Dulbecco’s modified Eagles medium (DMEM) (Invitrogen) containing 3.7 g/L sodium bicarbonate and supplemented with 10% heat inactivated fetal bovine serum (FBS) (Invitrogen). Cells were incubated at 37 °C and 5% CO_2_ in a humidified CO_2_ incubator.

### Cytotoxicity assay and Confocal imaging

Cytotoxicity assay of NZMOE:5% Li^+^ was performed using HepG2 cells and cell proliferation was measured by using WST-1 reagent (Roche). Briefly, HepG2 cells were harvested from cultures after brief trypsinization. Cells were seeded in 96 well plates at density of 3 × 10^3^ cells per well and incubated for 24 h. Solutions of various concentrations of compound were prepared by adding compound (in 0.08%HCl) to the fresh culture medium. Medium in culture wells was aspirated off and cells were treated with concentrations of 0, 5, 10, 20, 40, 80, and 160 μg/ml of NZMOE:5% Li^+^ in DMEM supplemented with 10% FBS for 24 h. After treatment, 10 μl of WST-1reagent was added in each well (containing 100 µl of medium) as per manufacturer’s instruction and incubated for 4 h. Then absorbance was measured at 440 nm (reference wavelength 700 nm) using Synergy HTX multimode reader (Biotek) and analysedwith Gen5 3.0 software. Wells containing cell culture medium with solvent (0.08%) were taken as blank. Percentage survival of cells was calculated as follows:

$${\rm{Viability}}( \% )=\frac{{[{A}]}_{{test}}}{{[{A}]}_{{control}}}\times 100$$

where, [A]_test_ is absorbance of test sample and [A]_control_ is absorbance of control (untreated) sample.

For Confocal Microscopy imaging, HepG2 cells were treated with 20 μg/ml of NZMOE:5% Li^+^ for 2 h at 37 °C under standard incubation conditions. After treatment, cells in 96-well plates were washed twice with 1x Phosphate buffer saline (PBS) and subjected to Laser Scanning Confocal Microscopy (A1, Nikon, Japan). The images were captured at 40x optical magnification with auto-exposure function of the instrument using by NIS-AR-SP NIS Element AR v1.0 software. The fluorescence of nanoparticles was visualized at excitation range 465–495 nm. The photomicrographs at single focus were captured under differential interference contrast (DIC) and fluorescence modes.

### Characterization Techniques

The phase of sample analyzed by Advanced D8 Bruker X-ray diffractometer (XRD) having Ni-filtered Cu-Kα (1.5405 Å) (2θ- 10-80° and step size 0.02°). The morphology of particles was determined by using transmission electron microscope (TECNAI G2). The Raman spectra of the samples were recorded with Renishaw micro-Raman spectrometer (laser excitation source of 633 nm). UV-Vis spectra were recorded using UV-2700 Double beam spectrophotometer in the absorbance mode. To measure absorbance, samples were dispersed in the methanol. Photoluminescence measurements of powder were carried out under ultraviolet excitation using 266 nm radiation from aNd: YAG laser and detected by a CCD (charge coupled device) detector (Model: QE 65000, Ocean Optics, USA) attached with the fiber. Lifetime decay was recorded with Edinburg instrument F-920 equipped with 100 W flash xenon lamp as the excitation source.

### Data analyses

The cell cytotoxicity data was presented as mean with standard deviation (SD). The data was analysedfor statistical significance by using Student’s t-test and p < 0.05 was considered as statistically significant.

## Results and Discussion

### Structural analysis

**Figure 1:**
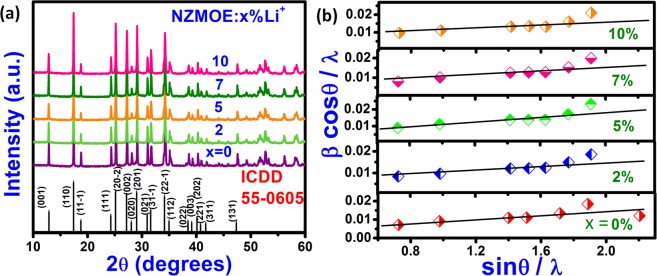
(**a**) XRD pattern and (**b**) Williamson Hall plot to measure micro-strain of Li^+^ (0, 2, 5, 7 and 10 at%) co-doped NZMOE phosphor.

**Figure 2:**
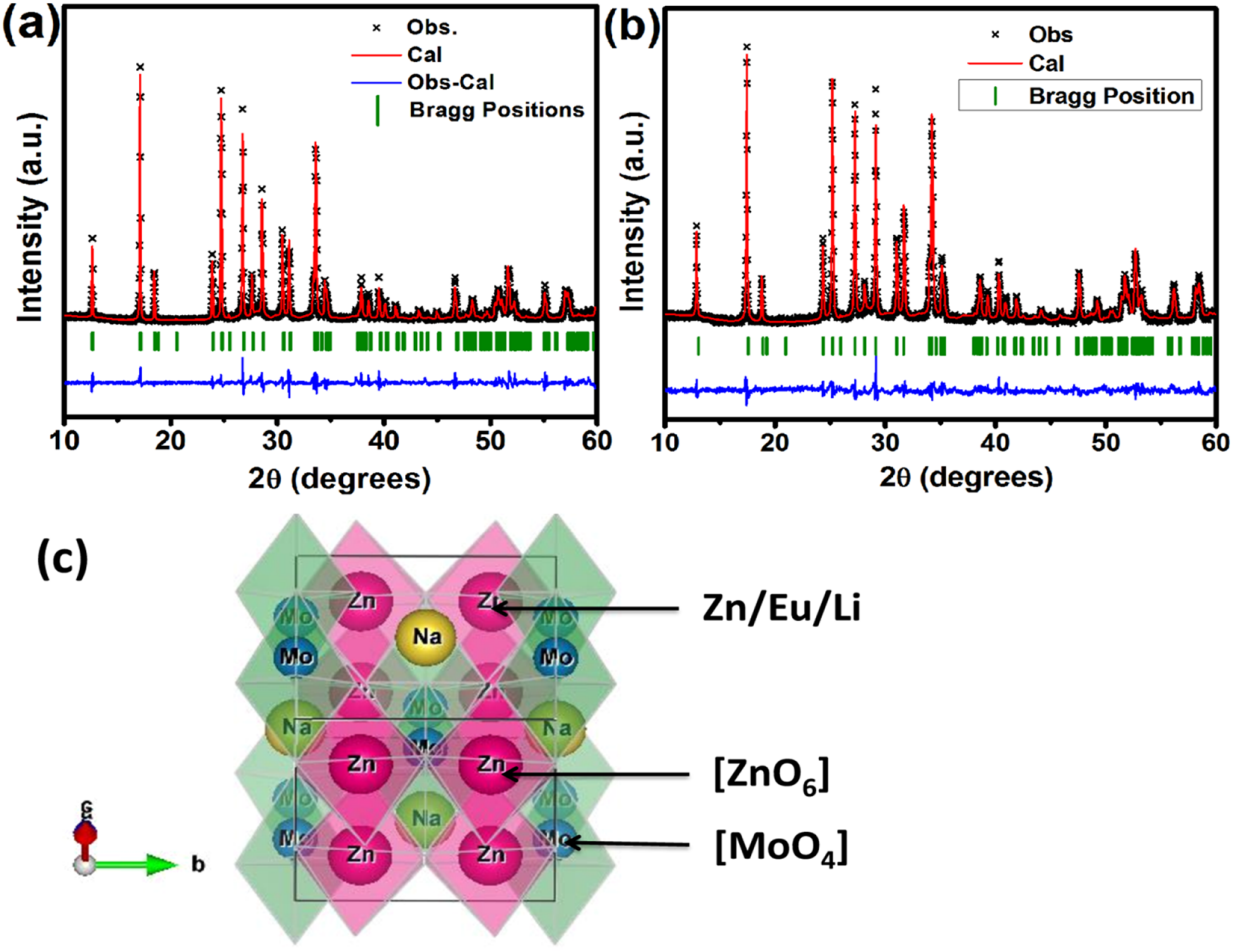
Reitveld Refinement plots of (**a**) NZMOE, (**b**) NZMOE: 5 at% Li^+^ and (**c**) schematic polyhedral representation of sodium zinc molybdate having [ZnO_6_] and [MoO_4_] clusters.

The phase of as prepared NZMOE: *x* at.% Li (*x* = 0, 2, 5, 7 and 10) samples was examined by XRD spectra. Figure 1(a) represents XRD pattern of Li^+^ co-doped sample. All diffraction peaks of as preparedLi^+^ co-doped phosphor matched with ICDD card no. 055-0605 have monoclinic phase with space group C 2/m of sodium zinc molybdenum oxide (Na(OH)Zn_2_(MoO_4_)_2_.2.5H_2_O). The diffraction intensity of peaks present in the range 30–33 °C were changes for Eu^3+^ doped and Li^+^ co-doped phosphor because Li^+^ ion affect arrangement of atoms in crystal so that intensity of diffracted rays from crystal were changed. The Rietveld refinement of as prepared Eu^3+^ activated phosphor was performed by using FullProf software. XRD pattern refined by using linear interpolation background type and peak shape was pseudo voigt. Temperature factor was taken anisotropic in Rietveld refinement. Figure 2(a,b) present Rietveld refinement plot of monoclinic phosphor for NZMOE and 5 at% Li^+^ co-doped NZMOE where χ^2^ is 4.08 and 1.97, R_wp_ is 14.4 and 11.6 and R_Bragg_ is 2.81 and 2.14, respectively. Refined parameters for 2 at% Eu^3+^ doped and 5 at% Li^+^ co-doped phosphor are given in Table 1. The data clearly shows that atomic positions of Zn and Na are fixed and there is minor shift in atomic positions of Mo metal observed with Li^+^ co-doping in NZMOE. However, atomic positions of O atom show remarkable changes owing to the fact that Li^+^ doping alters crystal environment. The shifting observed is because Eu^3+^ and Li^+^ ions takes Zn^2+^ site in crystal and Zn^2+^ is co-ordinated with oxygen atom. Metal to oxygen linkage length are also given in Table 1 which also attribute that Li^+^ incorporation in crystal owing to decrease in zinc to oxygen bond length. It might be because of smaller ionic size of Li^+^ ion so that bond lengths slightly shrink.

**Table 1:** Refined position of Zn, Mo, Na and O atom with their bond length (BL) between metal and oxygen linkage of 2% Eu^3+^ doped and 5% Li^+^ co-doped sodium zinc molybdate obtained by Reitveld refinement. (Standard deviation are given in bracket).

Atom	2% Eu^3+^			2% Eu^3+^, 5% Li^+^			Name of Atoms	2% Eu^3+^	2% Eu^3+^, 5% Li^+^
	*x*	*y*	*z*	*x*	*y*	*Z*		BL (Å)	BL (Å)
Zn	0.250 (00)	0.250 (00)	0	0.250 (00)	0.250 (00)	0	Zn—O2	2.273 (13)	2.183 (04)
Mo	0.079 (02)	0	0.289 (02)	0.079 (02)	0	0.289 (02)	Zn—O3	2.067 (05)	1.979 (14)
Na	0	0.500 (00)	0.500 (00)	0	0.500 (00)	0.500 (00)	Zn—O4	2.223 (14)	2.107 (02)
O1	0.238 (05)	0	0.518 (01)	0.235 (02)	0	0.538 (19)	Na—O1	2.575 (01)	2.644 (06)
O2	0.468 (04)	0.267 (07)	0.270 (10)	0.466 (02)	0.265 (09)	0.270 (10)	Na—O2	2.392 (05)	2.350 (09)
O3	0.156 (14)	0	0.080 (02)	0.175 (06)	0	0.090 (26)	Mo—O1	1.758 (02)	1.828 (09)
O4	0.630 (18)	0	0.094 (22)	0.644 (04)	0	0.081 (01)	Mo—O2	1.807 (05)	1.802 (05)
Eu/Li	0.250 (00)	0.250 (00)	0	0.250 (00)	0.250 (00)	0	Mo—O3	2.050 (22)	2.082 (24)

Figure 2(c) illustrates schematic polyhedral representation of monoclinic phase of NZMOE. It was modelled by VESTA Software and indicates high inversion symmetry. Figure 2(c) clearly reflects that each Zn^2+^ is coordinated to six oxygen atoms and forming octahedral [ZnO_6_] units^33,34^. Moreover, Mo^6+^ is coordinated to four oxygen’s having tetrahedral configuration [MoO_4_]^33^. It can also seen from structure that Na^2+^ is also surrounded by six oxygen atoms. In unit cell, oxygen works as bridge between zinc atoms as well as for Na and Mo atoms. It has layered structure containing eight zincs, four sodiums and ten molybdenums in a unit cell. The atomic radii are 0.74 Å for Zn^2+^, 0.95 Å for Eu^3+^ ion 1.02 Å for Na^+^, 0.59 Å for Mo^6+^ and 0.76 Å Li^+^. Since ionic radius of Eu^3+^ and Li^+^ and Zn^2+^ are nearly same Eu^3+^ and Li^+^ take Zn^2+^ site in crystal. Eu^3+^ concentration was constant so defects which were created by mismatching oxidation state of Eu^3+^ and Zn^2+^ should be constant. Li^+^ accommodate in these defects so Li^+^ increasing concentration reduces number of defects in crystal so that crystallinity was improved. Li^+^ co-doping must alter the lattice parameter and cell volume of NZMOE. The variations in such parameters are listed in Table 2 with the standard data. The data represents that cell volume of crystal enlarges with Eu^3+^doping due to larger ionic radius of Eu^3+^ ion (0.95 Å) compared to Zn^2+^ (0.74 Å). Furthermore, cell volume has shrunk with Li^+^ incorporation according to previous reported observations^30,31^. The contraction in cell volume occurs because Li^+^ ion takes interstitial site and it attracts oxygen^31^. The Scherer formula was used to estimate crystalline size which is given as

1 $${\rm{D}}=\frac{{\rm{0}}\mathrm{.89}{\rm{\lambda }}}{{\beta }\,\cos \,{\theta }}$$

**Table 2:** Lattice parameters, cell volume, crystalline size and strain of Li^+^ (0, 2, 5, 7 and 10 at.%) co-doped NZMOE (α = γ = 90°) obtained from Reitveld refinement.

Li+ (at.%)	Lattice parameters				Cell Volume (Å^3^)	Crystalline size (nm)	Micro-strain
	a (Å)	b(Å)	c (Å)	β (°)			
JCPDS 055–0605	9.453	6.340	7.642	115.89	412.1	—	—
0	9.630	6.401	7.504	116.14	415.26	48	0.00376
2	9.455	6.342	7.645	115.93	412.38	49	0.00347
5	9.457	6.342	7.645	115.94	412.30	52	0.00204
7	9.452	6.342	7.645	115.93	412.19	58	0.00282
10	9.455	6.341	7.644	115.93	412.14	66	0.00193

where, D is average crystalline size, λ is wavelength of X-rays (0.15405 nm), β is full width at half maximum and θ is the diffraction angle. Crystalline size was calculated by using 2θ values 17.42 (110), 25.18 (20$\bar{2}$), 27.23 (102) and 29.10 (201). From Table 1 it could be remarkable that crystalline size increases with Li^+^ incorporation. In addition, it is clear that Li^+^ incorporation distorts crystal structure parameters like bond length and bond angle.

However, change in bond length between two atoms creates strain into crystal. So, strain was calculated by Williamson Hall fitting method^35^. The equation for strain calculation is given as:

2 $$\frac{{{\beta }}_{{hkl}}\,\cos \,{\theta }}{{\lambda }}=\frac{1}{{{D}}_{{hkl}}}+{{\varepsilon }}_{{hkl}}\frac{\sin \,{\theta }}{{\lambda }}$$

where, β_hkl_ is full width at half maximum, θ is Bragg’s diffraction angles, ***D***_***hkl***_ is the effective crystalline site and ${{\boldsymbol{\varepsilon }}}_{{\boldsymbol{hkl}}}\,$is the micro-strain. The micro-strain estimated by slope of sinθ/λ Vs βcosθ/λ plot which is shown in Fig. 1b. The positive slope reflects that tensile strain is present into crystal which is also explaining expansion of cell volume with lithium incorporation^32^. The value of micro-strain for various compositions of Eu^3+^ and Li^+^ co-doped phosphor are summarized in Table 2. From Table 2, it could be inferred that the micro-strain is decreasing Li^+^ incorporation into NZMOE crystal. It supports our previous observation that cell volume is highest for 2 at% Eu doped nanoprobes so that microstrain is also maximum for the same^16^. Nonetheless, Li^+^ co-doped nanoprobes cell volume and microstrain decreases^30^.

### TEM and HRTEM analysis

**Figure 3:**
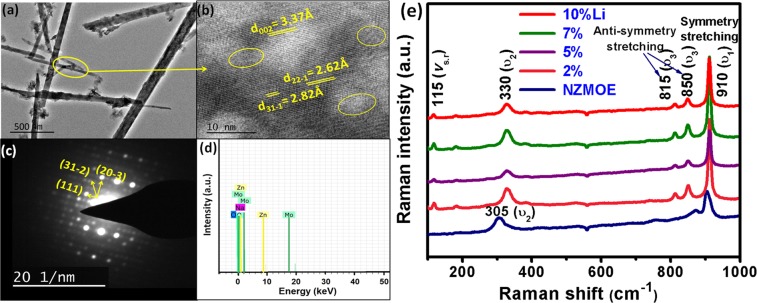
(**a**) TEM image, (**b)** corresponding HRTEM, (**c**) SADP, (**d**) EDX of 5 at% Li^+^ co-doped NZMOEand (**e**) Raman spectra of 0, 2, 5, 7, 10 at% Li^+^ co-doped NZMOE.

Figure 3(a–c) illustrates TEM (transmission electron microscopy), HRTEM (high resolution TEM) and selected area diffraction pattern (SADP) micrograph of 5 at% Li^+^ co-doped NZMOE phosphor. The TEM image shown in Fig. 3(a) indicates that morphology of monoclinic phosphor are rod like structure and its diameter is around 30–50 nm. The HRTEM of corresponding samples showed d-spacing of monoclinic phase (ICDD 055-0605). Although, there were defects in grain boundaries (mark with circle) were also observed in HRTEM. As it is known that defects or vacancies might create obviously affects on PL properties. Furthermore, SADP pattern shown in Fig. 3(c) shows SADP of as prepared sample having (hkl) in one direction is (111) and perpendicular to it is (20$\bar{3}$), the resultant of both planes are (31$\bar{2}$). Figure 3(d) showing EDX spectra of 5% Li^+^ co-doped NZMOE nanoprobes confirm presence of elements sodium, zinc, molybdenum and oxygen.

### Raman analysis

Monoclinic NZMOE vibrational modes have analysed by Raman Spectra which is shown in Fig. 3(e). From spectra, the vibrational modes are obtained at 330, 815, 850 and 910 cm^−1^. From earlier report, it is concluded that for monoclinic phase of zinc molybdate, vibrational peak in range 800–900 cm^−1^ should be found^34^. The vibrational modes in range 900–920 cm^−1^ and 780–880 cm^−1^ are due to symmetric and anti-symmetric stretching bond of tetrahedral O—Mo—O, respectively^36^. The other peaks centred at 305,330 and 115 cm^−1^ are observed due to symmetry stretch of Zn—O unit^24,34^. The slight shift in higher wave number side can be seen from Raman spectra which is due to change in bond length in crystal due to Li^+^ doping. It is supported from Rietveld refinement parameters obtained from XRD data. Additionally, with Li^+^ doping tensile strain creates which might be cause of shift in wave- number. The Raman spectra confirm presence of tetrahedral [MoO_4_] and octahedral [ZnO_6_] units.

### UV-Visible spectra

**Figure 4:**
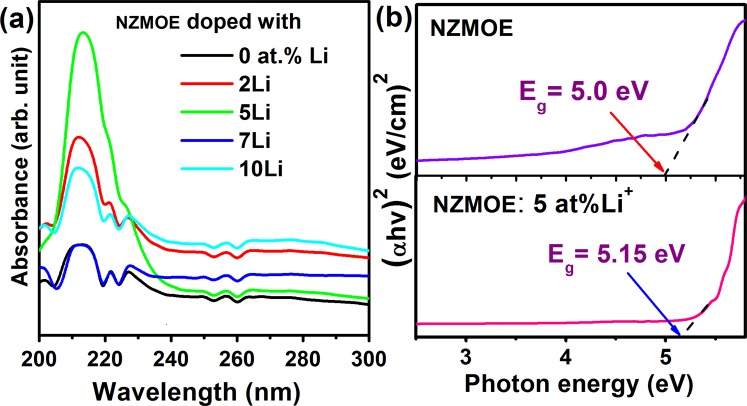
(**a**) UV-Visible absorption spectra and (**b**) Band gap determination via (αhν)^2^ versus photon energy plot for *x* at% Li^+^ (*x*- 0, 2, 5, 7 and 10 at%) co-doped NZMOE.

UV-Visible absorption spectra are shown in Fig. 4(a) for all Li^+^ co-doped nanoprobes. From spectra it is clearly shown that NZMOE strongly absorb in 210–230 nm range owing to charge transfer band (O^2−^ to Eu^3+^ ion). Absorption intensity increases with increasing Li^+^ concentration until 5% than decreases. The reason behind these variations is change in crystal parameters with Li^+^ incorporation as observed from XRD. If crystallinity gets improved than maximum incident photons were efficiently absorbed by the host NZMO. The band gap has been calculated by using wood and Tauc equation αhν = K ${(h{\boldsymbol{\nu }}-{{\boldsymbol{E}}}_{{\boldsymbol{g}}})}^{{\boldsymbol{n}}}$, where α -absorption coefficient, υ -frequency of absorbed photon, h -Planck constant, and ***E***_***g***_ -optical band gap^37^. The exponent n value depends on type of optical transition. From previous reports, it is known that molybdate family have direct allowed transition and for it n = ½^25,34^. The band gap measurement plot is shown in Fig. 4(b). It is 5 eV for NZMOE and 5.15 eV for 5% Li^+^ co-doped NZMOE. Band gap is slightly increase on Li^+^ co-doping supported with Kumar *et al*. observation in which band gap increased with Li^+^ co-doping in Gd_2_O_3_:Eu^3+^ ^31^. From above discussion, it could be conclude that with Li^+^ co-doping large absorption band was found which obviously helps to improved PL properties.

### PL Study

**Figure 5:**
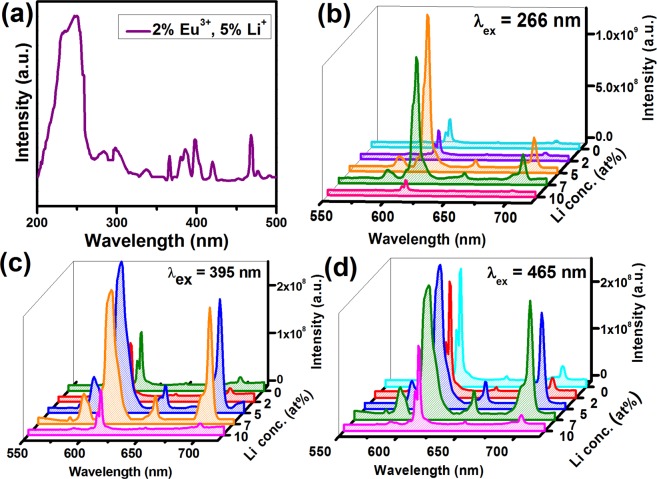
(**a**) PL excitation spectra of 5 at% Li^+^ co-doped NZMOE by monitoring 613 nm emission, and PL emission spectra of Li^+^ (0, 2, 5, 7 and 10 at%) co-doped NZMOE under (**b**) 266 nm, (**c**) 395 nm and (**d**) 465 nm excitation.

PL excitation spectra of 5 at% Li^+^ co-doped NZMOE are shown in Fig. 5(a). It consist a number of characteristic 4f-4f transition peaks of Eu^3+^ ion with host absorption broad band. The host absorption band is observed in 210–280 nm due to charge transfer transition of electron from O^2−^ (2P state) to Eu^3+^ (4f state) ions^32^. Simultaneously, other peaks due to characteristic excitation peaks of Eu^3+^ ions observed at 298, 336, 361, 383, 395, 415 and 466 nm corresponding to ^7^F_0_ → ^5^F_4_, ^7^F_0_ → ^5^H_3_, ^7^F_0_ → ^5^D_4_, ^7^F_0_ → ^5^G_2–6_, ^7^F_0_ → ^5^L_6_, ^7^F_0_ → ^5^D_3_ and ^7^F_0_ → ^5^D_2_, respectively^38^. Among various Eu^3+^ excitation peaks, the prominent intensity is found for 395 and 466 nm peaks. It illustrate that present phosphor could be efficiently absorbed by near ultra-violet and blue LED chip. This is the reason that in present report emission spectra have been recorded under 395 and 465 nm excitation wavelengths. Moreover, charge transfer from host [MoO_4_]^2−^ to Eu^3+^ happens at around 266 nm and emission spectra recorded at same wavelength^34^.

**Figure 6:**
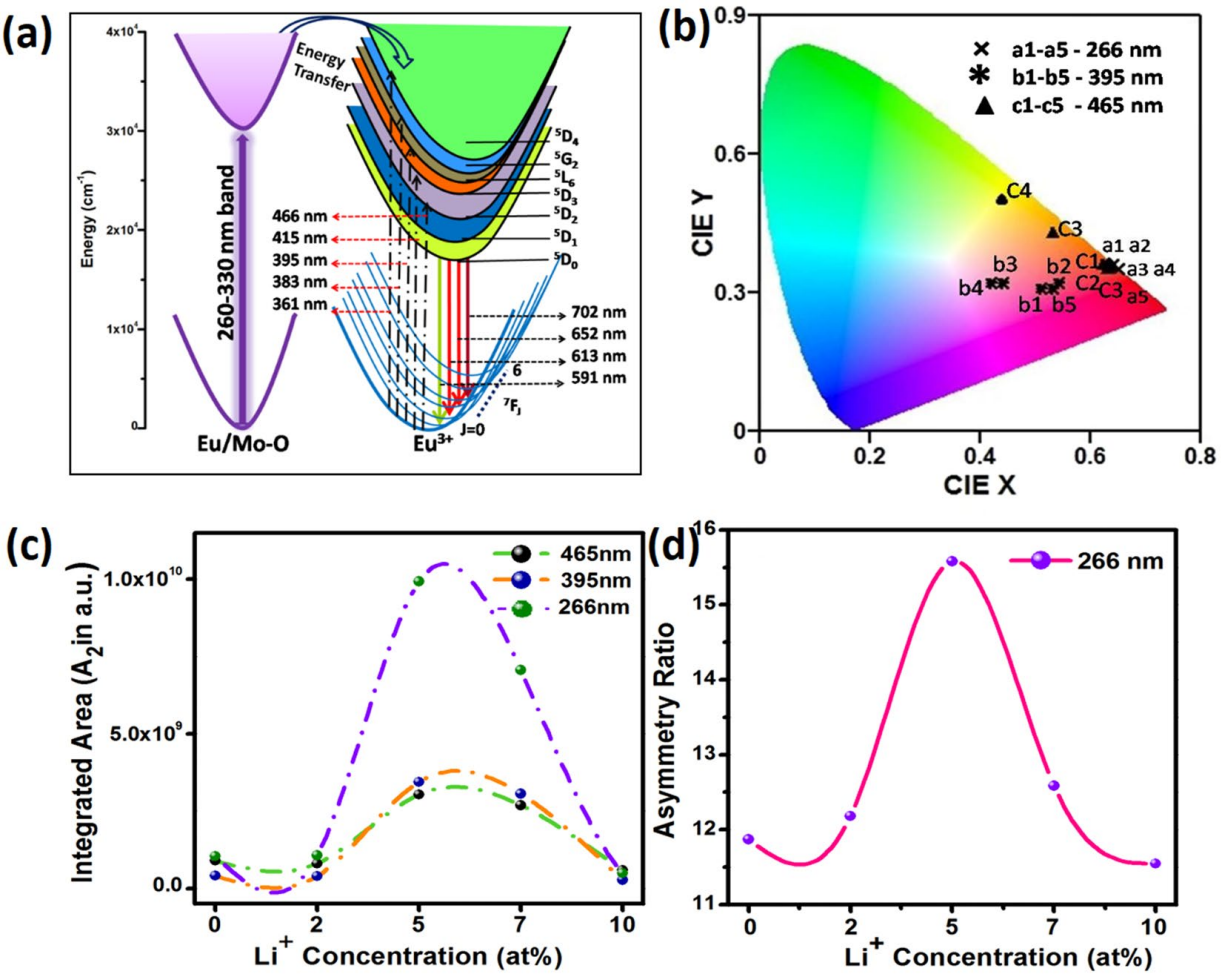
(**a**) Schematic representation of energy levels for energy transfer between MoO_4_^2−^ and Eu^3+^ ion with electronic transitions of Eu^3+^ ion, (**b**) CIE Chromaticity diagram of 0, 2, 5, 7 and 10 at% Li^+^ co-doped NZMOE (a1-a5, b1-b5 and c1-c5) at 266, 395 and 465 nm, (**c**) Integrated intensity plot of various Li^+^ (0, 2, 5, 7 and 10%) doped NZMOE and (**d**) asymmetry ratio (A_21_) at 266 nm excitation.

PL emission spectra of *x* at% Li^+^ (*x*- 0, 2, 5, 7 and 10 at%) co-doped NZMOE at 266, 395 and 465 nm excitation are given in Fig. 5(b–d). There are several peaks centered at 591, 613, 652 and 702 nm corresponding to ^5^D_0_ → ^7^F_1_, ^5^D_0_ → ^7^F_2_, ^5^D_0_ → ^7^F_3_ and ^5^D_0_ → ^7^F_4_ transitions, respectively. Most of these, intensity is prominent for ^5^D_0_ → ^7^F_2_ electric dipole transition. This is because Eu^3+^ ions electric and magnetic dipole transitions are depends on symmetry environment of Eu^3+^ ion in host matrix^29,32^. Furthermore, it is noticeable that with Li^+^ co-doping, rate of radiative relaxation increases which causes improvement in intensity^30,31^. There are several reasons possible behind improvement of intensity such as change in symmetry environment of Eu^3+^ ion in crystal, reduction of defects density, charge compensation and creation of oxygen vacancies into crystal^30,31^. In NZMOE, Eu^3+^ takes Zn^2+^ site and both differ in their oxidation state after doping of Eu^3+^ when three zinc ions leave its side than two europiumenter into crystal. However, one vacancy left inside the crystal which capture photons incident on it so that radiative emission intensity reduced. Although, Li^+^ doped into it than Li^+^ ion fill this vacancy so that the number of radiative emitted photons improved. The schematic representation of energy level diagram for energy transfer between [MoO_4_] group and Eu^3+^ ion are illustrated in Fig. 6(a). Simultaneously, Eu^3+^ absorb energy then its valence electron get excite to its various excite energy levels (^5^D_2,3,4_, ^5^L_6_, ^5^G_2–6_) than transit to its ^5^D_0_ metastable state via non-raditive relaxation (heating/vibration). Thereafter, electrons transit to ^7^F_J_ (J = 1, 2, 3, 4) state by emitting visible photons.

As co-ordination number of zinc ion in monoclinic phase is already discussed in XRD analysis part which plays an important role in PL emission properties. Probing in fluorescence observed with increases Li^+^ concentration because its incorporation creates oxygen vacancies in crystal which could improve radiative transitions. Therefore, profound red emission observed for 5% Li^+^. But after optimum concentration, oxygen vacancies cause of reduction in intensity^31^. This is why that PL intensity increases until 5 at% Li^+^ concentration. After that, intensity starts decreasing because of increasing non-radiative relaxation rate due to defects in crystal and oxygen vacancies. In addition, the intensity of electric dipole emission also depends on excitation wavelength. Figure 6(c) represents integrated intensity of Li^+^ (0, 2, 5, 7 and 10 at%) co-doped NZMOE nanoprobes. From figure it is remarkable that highest integrated intensity observed at 266 nm excitation than the other excitations. It owing to efficient energy transfer from ligand [MoO_4_]^2−^ to Eu^3+^ ion. Here, host have two [MoO_4_]^2−^ ligand so it absorbs maximum incident photons as observed from UV-Visible absorption spectra and PL excitation spectra so that more Eu^3+^ ions get excite via charge transfer mechanism. As previously discussed, that intensity of electric dipole transition depends on asymmetry of Eu^3+^ ion in crystal environment. To evaluate asymmetry of Eu^3+^, the ratio of electric to magnetic dipole transition which is known as asymmetry ratio is calculated by following equation:

3 $${{\rm{A}}}_{21}=\frac{{\int }_{600}^{630}\,I\,d\lambda }{{\int }_{580}^{600}\,Id\lambda }$$

where A_2_ is the integrated intensity of ^5^D_0_ → ^7^F_2_ transition and A_1_ is integrated intensity of ^5^D_0_ → ^7^F_1_ transition, the variations in asymmetry ratio with Li^+^ co-doping in NZMOE are presented in Fig. 6(d). It showed A_21_ is highest for 5at% Li^+^ co-doped nanoprobes.

In 1931, the Commission International de-I’ Eclairage (CIE) chromaticity diagram developed which shows lighting color of phosphor. The CIE diagram for Li^+^ co-doped NZMOE is representing in Fig. 6(b). From figure, it is clear that with Li^+^ co-doping multi-color emission has been observed. However, it also depends on excitation wavelength such as for 266 nm excitation, CIE co-ordinate found in dark red region. These co-ordinates are much closer to National Television Standard Committee (NTSC) co-ordinate. Simultaneously, for 395 and 465 nm excitation CIE co-ordinate tune from pink/yellow to red region, respectively. Color tuning with Li^+^ co-doping and excitation wavelength is according to Parchur *et al*. observations^39^. Li^+^ co-doping creates change in environment of Eu^3+^ ion and alter various crystal parameters so that the intensity of magnetic and electric dipole transition is affected. Mixing of these two emission intensity gives various colour like from yellow, red and pink. The color purity for red region is calculated by using given formula^16,40^

4 $${\rm{Color}}\,{\rm{purity}}=\frac{\sqrt{{({\rm{x}}-{{x}}_{{i}})}^{2}+{({\rm{y}}-{{y}}_{{i}})}^{2}}}{\sqrt{{({{x}}_{{d}}-{{x}}_{{i}})}^{2}+{({{y}}_{{d}}-{{y}}_{{i}})}^{2}}}\times 100 \% $$

where (*x, y*), (***x***_***d***_, ***y***_***d***_) and (${{\boldsymbol{x}}}_{{\boldsymbol{i}}}$, ${{\boldsymbol{y}}}_{{\boldsymbol{i}}}$) are the co-ordinate of sample point, dominant wavelength and white illumination in CIE diagram, respectively. In general, taking prevailing CIE co-ordinates (${{\boldsymbol{x}}}_{{\boldsymbol{d}}}$, ${{\boldsymbol{y}}}_{{\boldsymbol{d}}}$) = (0.67, 0.32) and (${{\boldsymbol{x}}}_{{\boldsymbol{i}}}$, ${{\boldsymbol{y}}}_{{\boldsymbol{i}}}$) = (0.3101, 0.3162). With the help of these co-ordinates, the colour purity of Li^+^ co-doped phosphor are given in Table 3 which shows that red colour purity is more than 90% under 266 nm excitation. The color purity is utmost (94.9%) for more than 5 at% Li^+^ incorporation however for without Li^+^ co-doped phosphor it is only 92.4%. It is higher than recently reported color purity of ZrO_2_: Eu^3+^, Li^+^ (86%), Sr_0.8_Li_0.2_Ti_0.8_Nb_0.2_O_3_: Eu^3+^ (94.7%), CaW_0.4_Mo_0.6_O_4_:Eu^3+^ (93.8%)^30,41,42^. Although, for other excitation wavelength (395 and 465 nm), red color purity is decreased because for these excitations intensity of electric dipole is not much high as compared to magnetic dipole transitions. Additionally, color correlated temperature (CCT) estimated using McCany empirical formula are given in Table 3 ^43^. Its values fall in the range 1600–5500 K for different excitation wavelengths (266, 395 and 465 nm).

**Table 3:** CIE Chromaticity co-ordinate, color purity and color correlated temperature (CCT) of Li^+^ co-doped (0, 2, 5, 7 and 10 at.%) NZMOE.

*λ*_*ex*_	Li^+^ conc. (at.%)	CIE co-ordinate	Color Purity (%)	CCT (K)
266 nm	0	(0.64, 0.36)	92.4	1916
	2	(0.64, 0.36)	92.4	1916
	5	(0.65, 0.35)	94.9	2152
	7	(0.65, 0.35)	94.9	2152
	10	(0.65, 0.35)	94.9	2152
395 nm	0	(0.51, 0.31)	55.6	1634
	2	(0.54, 0.32)	63.9	1691
	5	(0.44, 0.32)	36.1	2148
	7	(0.33, 0.35)	30.5	5440
	10	(0.53, 0.32)	61.1	1651
465 nm	0	(0.62, 0.36)	86.9	1788
	2	(0.63, 0.35)	89.4	1973
	5	(0.53, 0.43)	68.8	2104
	7	(0.44, 0.50)	62.5	3601
	10	(0.63, 0.36)	89.7	1848

**Figure 7:**
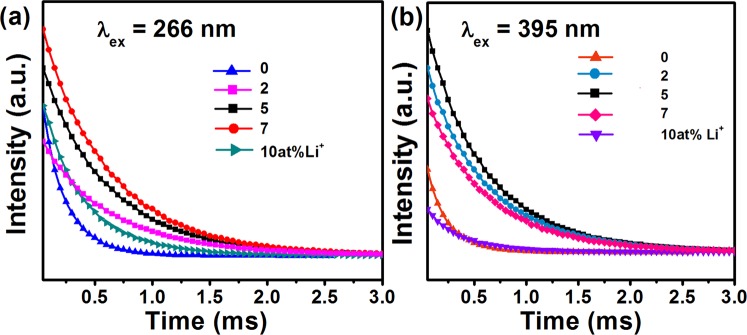
Decay profile of Li^+^ (0, 2, 5, 7 and 10%) doped NZMOE under (**a**) 266 and (**b**) 395 nm excitation, respectively by monitoring electric dipole ^5^D_0_ → ^7^F_2_ transition (613 nm).

The decay profile was recorded by monitoring 613 nm (^5^D_0_ → ^7^F_2_) emission of Eu^3+^ ion under 266 and 395 nm excitations shown in Fig. 7(a,b), respectively. The decay curve is fitted by using mono-exponential fitting equation I (t) = I_0_exp $(\frac{-t}{\tau })$ where I_0_ is intensity of monitored emission at t = 0, I(t) is intensity at time t and τ is the lifetime of the emission. It is summarized in Table 4 (abbreviated as ${\tau }_{obs}$) for Li^+^ co-doped NZMOE at 266 nm excitation. Additionally, lifetime for 0, 2, 5, 7 and 10 at% Li^+^ co-doped NZMOE at 395 nm excitation were found 0.3390, 0.5767, 0.57736, 0.5662 and 0.3982 ms, respectively. From these observations, it is remarkable that with Li^+^ incorporation lifetime are increased because of increasing rate of radiative and non-radiative transitions.

**Table 4:** Radiative parameters viz radiative transition rate (A_R_), lifetimes (τ_obs,_ τ_rad_), Intensity parameters (Ω_2,_ Ω_4_), stimulated cross section σ_*λp*_, branching ratio *β*_o*J*_ and quantum efficiency (η) of Li^+^ (0, 2, 5, 7 and 10%) doped NZMOE (at λ_*ex*_ = 266 nm).

Li^+^ (at.%)→	0	2	5	7	10
A_R_ (s^−1^)	781	1084	1189	1005	918
τ_rad_ (ms)	1.28	0.92	0.84	0.99	1.09
τ_obs_ (ms)	0.41	0.57	0.59	0.60	0.52
Ω_2_ (×10^−19^ cm^2^)	2.015	2.815	3.277	2.708	2.492
Ω_4_ (×10^−19^ cm^2^)	0.392	0.315	0.420	0.398	0.301
η (%)	31.77	61.93	70.02	60.20	48.18
β_01_ (%)	6.40	4.61	4.20	4.98	5.45
β_02_ (%)	84.63	84.54	89.42	87.68	88.41
β_04_ (%)	7.95	9.46	5.61	6.26	5.19
σ_01_ (×10^−20^ cm^2^)	0.491	0.72	0.798	0.583	0.529
σ_02_ (×10^−20^ cm^2^)	1.702	1.06	2.627	2.176	2.027
σ_04_ (×10^−20^ cm^2^)	1.748	2.276	2.803	2.591	2.108

### Judd Ofelt (J-O) Analysis and Radiative Properties

To explain PL emission and lifetime measurements, Judd Ofelt (J-O) parameters have been calculated which give information about local site symmetry of Eu^3+^ ion, crystal field strength of host and bond vicinity of rare earth ion with host^29^. It also gives information about spectral behaviour of rare earth ion and determines the rate of radiative and non-radiative (NR) transition rate as well as quantum efficiency^30,31,44^. There are three Judd Ofelt parameters Ω_λ_(λ = 2, 4 and 6) which can determine rate of radiative transitions of forbidden electric dipole transitions (^5^D_0_ → ^7^F_J_, J = 2, 4, 6). It helps to identify asymmetry of Eu^3+^ ion into host matrix with Li^+^ incorporation. Among various parameters calculated by J-O theory, spontaneous emission rate of magnetic dipole transition A_01_ taken is 50 S^−1^ ^30,39^. In general for other ^5^D_0_ → ^7^F_J_ (J = 2, 4 and 6) transitions of Eu^3+^ ion, radiative transition rate could be esteemed by calculating with formula^29^

5 $${{\rm{A}}}_{0{\rm{J}}}=\frac{64{\pi }^{4}{\nu }_{J}^{3}n{({n}^{2}+2)}^{2}}{3h(2J+1)\,9\,}{{\rm{e}}}^{2}\sum _{J=2,\,4,\,6}\,{\Omega }_{\lambda }| < {}^{5}{\rm{D}}_{0}\Vert {{\rm{U}}}^{(\lambda )}\Vert {{}^{7}{\rm{F}}_{{\rm{J}}} > \Vert }^{2}$$

where, n is refractive index of given phosphor (1.84) and ν_1_ is the wavenumber of ^5^D_0_ → ^7^F_1_ emissive transition (16949 cm^−1^). Consequently, ν_J_ represents wavenumber of ^5^D_0_ → ^7^F_J_ (J = 2, 4, 6) transitions, respectively, e is the electronic charge and h is Planck’s constant. In current experiment J = 6 transition is absent because it is found in IR region and can’t be determine due to instrument limit.

The matrix element |<^5^D_0_||U^(λ)^|| ^7^F_J_>||^2^ is 0.0035, 0.0030 for J = 2, 4, respectively. From PL emission spectra, it is known that among various characteristic emission peaks of Eu^3+^ ion, the intensity of 613 nm is dominant. Through these observations, it is concluded that with Li^+^ incorporation high inversion asymmetry of Eu^3+^ ion occurs into sodium zinc molybdate crystal. In J-O theory Ω_2_ represents intensity of electric dipole transition and it should be more as compared to other intensity parameter. Meanwhile, on the other hand according to this theory ^5^D_0_ → ^7^F_J_ transitions are magnetically and electrically forbidden for J = 0, 3, 5. Thus, the matrix elements related to these transitions are zero. Nonetheless, weak intensity has been observed for J = 3 because of induced crystal field J-mixing. The total radiative transition probability is calculated by addition of all individual radiative transition probability. It could be write in equation form as^31^

6 $${A}_{R}=\sum _{J}\,{A}_{0J}={A}_{01}\frac{{\nu }_{01}}{{I}_{01}}\sum _{J=0}^{4}\,\frac{{I}_{OJ}}{{\nu }_{0J}}$$

where ${\nu }_{01}$ and ${\nu }_{0J}$ are wavenumber again *I*_01_ and *I*_0*J*_ are integrated intensity of ^5^D_0_ → ^7^F_1_ and ^5^D_0_ → ^7^F_J_ (J = 2, 4) transitions, respectively. The reciprocal of radiative transition rate gives radiative decay lifetime and it can be expressed in mathematical form:

7 $${\tau }_{rad}=\frac{1}{{A}_{R}}=\frac{1}{{\sum }_{J}\,{A}_{0J}}$$

However, total transition rate is sum of radiative and non-radiative transitions rates. It is found from reciprocal of experimentally observed lifetime and it can be estimated in equation form as

8 $${A}_{T}={A}_{R}+{A}_{NR}=\frac{1}{{\tau }_{obs}}$$

Stimulated emission cross section (*σ*_*λp*_) used to measure laser transition in any emission process. Higher value of *σ*_*λp*_ indicatehigher rate of stimulated emission. It can be calculated by using following equation:

9 $${\sigma }_{\lambda p}=\frac{{\lambda }_{p}^{4}}{8\pi c{n}^{2}{\rm{\Delta }}{\lambda }_{eff}}{A}_{T}$$

Additionally, fluorescent branching ratio has been calculated to measure relative intensity of particular transition (^5^D_0_ → ^7^F_J_, J = 1, 2, 4) with respect to total radiative transition. It is expressed by equation

10 $${\beta }_{0J}=\frac{{A}_{0J}}{\sum {A}_{0J}}$$

Furthermore, Judd Ofelt theory also gives a formula to calculate quantum efficiency of phosphor. It is the ratio of number of photons emitted to the number of photons absorbed by the phosphor in luminescence process. From J-O theory, quantum efficiency (QE) of a phosphor material in PL emission process calculated with the help of ratio of observed lifetimes to the radiative transitions lifetime. It is denote by η and given by formula

11 $${\rm{\eta }}=\frac{{\tau }_{obs}}{{\tau }_{rad}}$$

Therefore, from equation J-O parameters such as *A*_*R*_, ${\tau }_{obs}$, ${\tau }_{rad}$, ${{\rm{\Omega }}}_{2}$, ${{\rm{\Omega }}}_{4},{\sigma }_{0J}$, ${\beta }_{0J}\,$and η are calculated and summarised in Table 4. The variation in J-O parameters observed due to Li^+^ incorporation. Fluorescent branching ratio (${\beta }_{0J}$) increases with Li^+^ co-doping for electric dipole transitions and higher than recently reported its values by Loiko *et al*.^45^. In addition, with increasing Li^+^ concentration quantum efficiency significantly enhanced and extreme for 5 at% Li^+^ co-doped NZMOE (70%). Further increasing Li^+^ content it is decreased and it might be due to excess vacancy of oxygen atom. The higher concentration of Li^+^ ion also causes concentration quenching and surface defects. Nonetheless, QE of presently reported phosphor is high enough rather than currently reported QE by Prakashbabu *et al*. and Jyothi *et al*.^30,41^. Again, ${\Omega }_{2}$ is also maximum for 5 at% Li^+^ co-doped monoclinic as prepared phosphors which denotes high asymmetry of Eu^3+^ in host matrices^46,47^. It can also be observed from Table 4 that ${\Omega }_{2}$ is least for without Li^+^ co-doped sample and higher for other Li^+^ co-doped sample which confirms that Li^+^ co-doping increases possibility of Eu^3+^ occupation on non-inversion symmetry sites.

### Cytotoxicity

**Figure 8:**
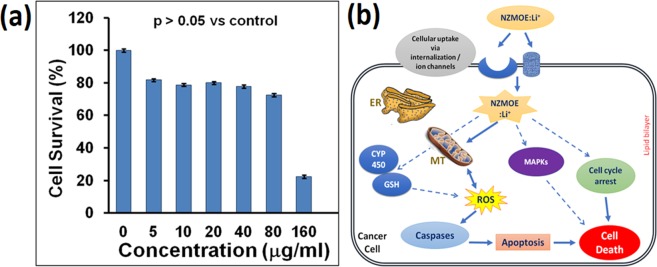
(**a**) Cell viability of HepG2 treated for 24 h with various concentration of NZMOE: 5% Li^+^ (0, 5, 10, 20, 40, 80, and 160 μg/ml). *p = 0.001 vs. Control, (**b**) Schematic representation of NZMOE:5% Li^+^ conjugation with HePG2 cells.

From PL spectra, it was evident that present phosphor material has good color purity and quantum efficiency and thus may be suitable for bio-imaging purposes. Therefore, the effect of the compound on cell viability (cytotoxicity) was measured to confirm the suitability of thecompound for biological assays. Cytotoxicity assessment is presented in Fig. 8(a) which indicates cell viability (%) of HepG2 cells treated with various concentrations of phosphor materials for 24 h. The viability of cells without treatment (vehicle control) was considered as 100%. From Fig. 8(a), it is evident that nearly 80% cells were viable after treatment with 5–80 μg/ml NZMOE:5% Li^+^ for 24 h. However, cells viability was reduced to 30% after treatment with160 μg/ml of the compound for 24 h. The high cytotoxicity observed at 160 µg concentration might be due to very high concentration of compound causing severe effects on cell growth. The statistical analysis of the data shows that the changes in viability at concentrations of 5 to 80 µg/ml remained non-significant (p > 0.05). While concentration of 160 µg/ml was statistically significant (p = 0.001) when compared with control untreated cells. LD_50_ (Lethal Dose)was calculated to be 126 μg/ml for NZMOE:5% Li^+^. LD_50_ represents the measurement of toxicity of any compound and it is the amount of tested material causing death of 50% cells. These data support our notion that NZMOE:5% Li^+^ may be used for biological imaging applications because of its low cytotoxicity.

### Confocal imaging

**Figure 9:**
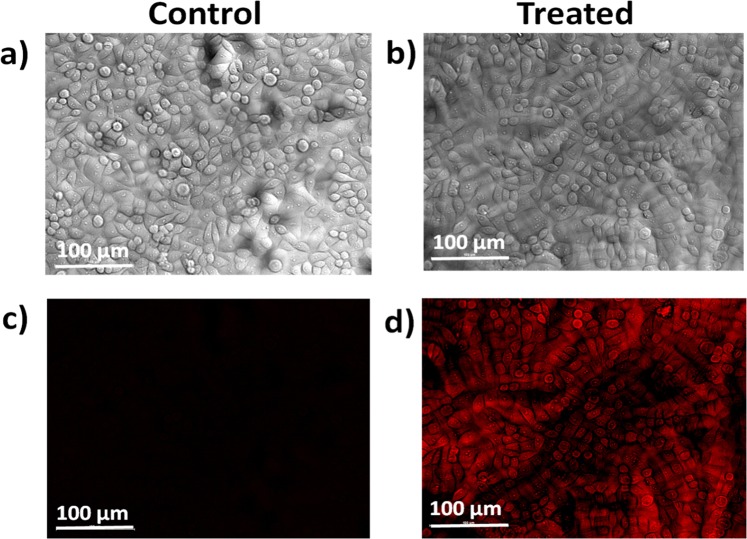
(**a,c**) Confocal imaging of HepG2 cells control and (**b,d**) treated cells with NZMOE:5% Li^+^ (red channel, 20 μg/ml) for 2 h. Upper panel is showing DIC images while lower panel is showing their respective florescent images at 40x magnification. The scale bar represents 100 µm size.

Since cytotoxicity assay confirmed the suitability of the phosphor material for biological imaging applications, we next examined whether the phosphor material is fluorescent inside cells^48,49^. Recently, Guo *et al*. reported that the luminescence intensity of tungstate phosphor is better than fluoride host. Similarly, in present work we report molybdate phosphor in which [MoO_4_]^2−^ efficiently transfer its energy to Eu^3+^ ion so its PL performance should be better than fluoride host^46^. For this purpose, confocal microscopy imaging of HepG2 cells after treatment with NZMOE:5% Li^+^ was performed. Figure 9(a–d) represent photomicrographs of differential interference contrast (DIC) and Confocal fluorescent images of HePG2 cells treated with 20 μg/ml concentration of NZMOE:5 at% Li^+^ for 2 h. The DIC image clearly represents those cells in control wells are viable and show negligible fluorescence under used excitation wavelength. While cells treated with 20 μg/ml of phosphor material showed no noticeable change in cell growth with bright red fluorescence which indicated that phosphor maybe a promising tool for bio-imaging purpose. The image (Fig. 9d) demonstrate that NZMOE:5 at% Li^+^ could enter inside cells through plasma membrane and gets localized in cytoplasm. Localization of NZMOE:5% Li^+^ in cytoplasm might explain the concentration dependent cytotoxicity as observed in Fig. 8(a). NZMOE:5% Li^+^ could accumulate in HepG2 cells till concentration 80 µg/ml with approximately 23% cytotoxicity (Fig. 8a) while it caused high cytotoxicity (70%) at 160 µg/ml concentration possibly because the HepG2 cells could not further accumulate NZMOE: 5 at% Li^+^ in cytoplasm. The conjugation mechanism of HePG2 cells with NZMOE: 5 at% Li^+^ fluorophore is given in Fig. 8(b). Based upon available literature and our observations, it is proposed that NZMOE:5 at% Li^+^ when entered in cytoplasm produced fluorescence as confirmed by confocal microscopy (Fig. 9(a–d)). Reported literature suggests that NZMOE: 5 at% Li^+^ might interact with Glutathione oxidative enzyme system and modulate free radical generation inside cells. This might lead to induction of apoptosis causing cell death at higher concentration. Likewise, NZMOE might modulate expression of MAPKs (Mitogen-activated protein kinases) that imparts cell survival and cell cycle progression. Cytochrome P450 appears to play important role in deciphering the effects of NZMOE in HepG2 cell proliferation. These studies reveal that synthesized nanoprobes produce deep red fluorescence with HePG2 cells.

## Conclusions

Li^+^ co-doped NZMOE having rod-like structures have successfully been synthesized by Polyol method. The XRD observations indicate a significant improvement in crystallinity and phase purity with Li^+^ co-doping. It also reveals a remarkable shift in the lattice parameters and cell volume. The optical performances of Li^+^ co-doped NZMOE nanoprobes have been determined from UV-VIS absorption spectra and PL spectra. High red color purity (94.9%) has been observed with Li^+^ co-doping at 266 nm excitation. Moreover, intensity and radiative transition parameters have been calculated by employing the J-O theory. The quantum efficiency has its extreme value for 5 % Li^+^ co-doped phosphor (70%) whereas quantum efficiency for without Li^+^ co-doped it is only 31.77% at excitation wavelength 266 nm. This bright red fluorescence obtained through Li^+^ ion incorporation suggests that the material could be a prominent phosphor material for optoelectronic devices, sensors and biological imaging. Cytotoxicity assay has been performed on HepG2 liver carcinoma cells and the respective LD_50_ was found to be around 126 μg/ml for 5% Li^+^ co-doped nanoprobes. It showed that present phosphor is biocompatible up to 80 μg/ml. Furthermore, confocal imaging of HepG2 cells treated with phosphor material indicates that the synthesized phosphor generates bright red fluorescence even at low concentration of sample (20 μg/ml) when entered in the cytoplasm. It attributes to the fact that the present double molybdate gives deep fluorescence and could be beneficial for bio-imaging purposes.
